# The incidence and prevalence of upper tract urothelial carcinoma: a systematic review

**DOI:** 10.1186/s12894-021-00876-7

**Published:** 2021-08-17

**Authors:** Ahmed Soualhi, Elke Rammant, Gincy George, Beth Russell, Deborah Enting, Rajesh Nair, Mieke Van Hemelrijck, Cecilia Bosco

**Affiliations:** 1 University Hospital Hairmyres, Glasgow, UK; 2 Translational Oncology and Urology Research, School of Cancer and Pharmaceutical Sciences, King’s College London, London, UK; 3 Department of Human Structure and Repair, Ghent University, Ghent, Belgium; 4 Department of Oncology, Guy’s Hospital, Guy’s and St Thomas’ NHS Foundation Trust, London, UK; 5 Department of Urology, Guy’s Hospital, Guy’s and St Thomas’ NHS Foundation Trust, London, UK

**Keywords:** Epidemiology, Incidence, Upper tract urothelial carcinoma, Urological oncology

## Abstract

**Background:**

Upper tract urothelial carcinoma (UTUC) is a rare urological cancer that is still an important public health concern in many areas around the world. Although UTUC has been linked to a number of risk factors, to our knowledge no systematic review has been published on the overall incidence and prevalence of de-novo UTUC. This review aimed to examine the global epidemiology of UTUC to provide clinicians and public health specialists a better understanding of UTUC.

**Methods:**

A systematic search was conducted on MEDLINE, Embase, and the Web of Science using a detailed search strategy. Observational epidemiological studies describing the incidence and prevalence of de-novo UTUC in adults were included, and the Joanna Briggs Institute checklist was used for critical appraisal and data extraction of the studies selected.

**Results:**

The systematic search identified 3506 papers, of which 59 papers were included for qualitative synthesis. The studies selected included data ranging from the years 1943 to 2018. A comprehensive qualitative synthesis of the data was performed. UTUC incidence generally varied according to age (higher with increasing age), sex (unclear), race (unclear), calendar time (increased, stable, or decreased according to region), geographical region (higher in Asian countries), occupation (higher in seamen and printers), and other population characteristics. Prevalence was only reported by one study, which showed UTUC to have the highest incidence of the rare urogenital cancers in Europe.

**Conclusion:**

This systematic review highlights an increased incidence of UTUC in certain groups, including increasing age and certain occupations such as seamen. The incidence of UTUC also varies between certain geographical regions. The trend of UTUC incidence for sex, race, and calendar time is less clear due to a wide variety of metrics used by the studies identified. More studies are also required on the prevalence of UTUC to understand its disease burden.

*Trial registration *This review was registered on PROSPERO (registration number CRD42019134255).

**Supplementary Information:**

The online version contains supplementary material available at 10.1186/s12894-021-00876-7.

## Background

Upper tract urothelial carcinoma (UTUC) is a relatively rare cancer of the urinary tract. UTUC describes a cancer originating in the renal pelvis, renal calices, and the ureter. Smoking and aristolochic acid are the two most common risk factors for UTUC [1, 2]. UTUC has been genetically linked to hereditary non-polyposis colorectal cancer (HNPCC), also known as Lynch syndrome, through mutations in DNA mismatch repair genes [3]. The link between UTUC and bladder cancer has also been studied, with both cancers being called ‘disparate twins’ for their similarities in histology and origins yet differences in diagnosis and treatment [4].

Understanding the epidemiology of UTUC can help aid clinical diagnosis and can be used to highlight high-risk groups that can be targeted with strategies to prevent such groups from developing UTUC [5]. However, whilst systematic reviews on the epidemiology of other urological cancers have been performed [6, 7], to our knowledge a systematic review of the incidence and prevalence of de-novo UTUC has never been published. The incidence of UTUC has been estimated at 1–2 cases per 100,000 [8], although this varies between age, geographical region, occupation, and other factors. This paper aims to provide a more comprehensive assessment of the global incidence and prevalence of UTUC in order to support guidance for future studies on its epidemiology.

## Methods

A detailed search strategy (Additional file 1) was developed using the Peer Review of Electronic Search Strategies (PRESS) checklist. Three databases were searched for this systematic review: MEDLINE, Embase, and the Web of Science Core Collection. No limitations on dates were applied, and the final search was done on 4th February 2021. This review was registered on PROSPERO (Registration Number CRD42019134255).

Observational studies (cross-sectional and cohort) describing the incidence and prevalence of de-novo UTUC in adults (≥ 18 years of age) were included. Studies that reported both lower and upper urinary tract carcinomas were only included if data for UTUC was separately reported. No further exclusion criteria were set for study setting and demographic factors. Studies were excluded if no full text was available, or if the abstracts were in any other language than English. There was no language restriction for full texts; translations were carried out if necessary. The reference lists of the papers selected were manually searched for further relevant studies to include in the data extraction.

Results from the database searches were managed in Mendeley and imported into Rayyan QCRI [9], where two independent authors (AS and ER) screened the titles and abstracts. Any remaining conflicts were resolved by a third author (CB). Both authors (AS and ER) then screened the full texts of the selected papers for relevance and bias using the Joanna Briggs Institute (JBI) Checklist for Prevalence Studies [10]. Any further conflicts were resolved by a third author (CB).

Data extraction from the selected papers was performed using the JBI Checklist and managed using Microsoft Excel. The following data were extracted: study title, study author(s), year of publication, journal, country, aim of study, population, setting, study design, study duration, outcomes measured, diagnostic criteria used, ethical approval, methods of data analysis, incidence (and 95% confidence interval), and prevalence (and 95% confidence interval).

Some papers did not report incidence data in numbers, instead showing only a graph. The authors of these studies were contacted for raw data, which are included in this review. Where raw data was unavailable, data from graphs in the selected articles were extracted using WebPlotDigitizer, a software tool that allows reverse data extraction from both linear and logarithmic graphs [11].

The data from the selected studies was found to be heterogeneous and hence a meta-analysis could not be conducted. Therefore, a narrative synthesis of the data was done according to the following subgroups: age, sex, race, geographic region, calendar time, occupation, Lynch syndrome, and other population characteristics.

## Results

**Fig. 1:**
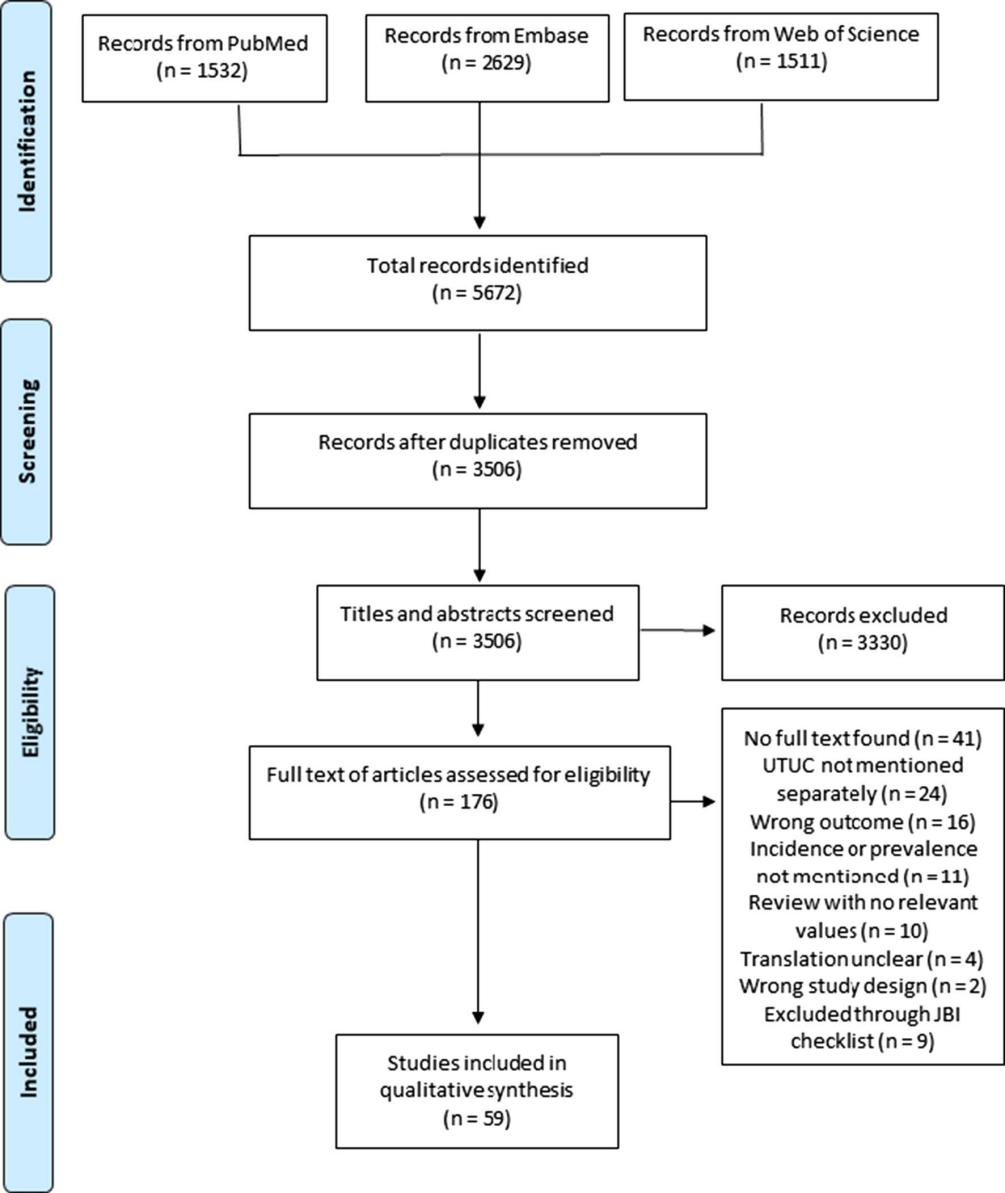
PRISMA flow diagram

The results of the database searches, title and abstract screening, and full-text screening are outlined in Fig. 1. The search strategy identified 3506 papers after duplicates were removed. After title, abstract, and full-text screening, 117 titles were excluded for a number of reasons, and 59 papers were included for this review (Table 1).

**Table 1:** Study descriptives of all studies selected

Study	Country	Setting	Duration	Factors reported
Noone et al. [35]	USA	SEER Registry	1992–2013	Race
Yu et al. [64]	South Korea	Samsung Medical Center	1995–2016	Overall incidence
Chernozemsky et al. [47]	Bulgaria	27 villages in the Vratza district	1965–1974	Calendar time
Kockelbergh et al. [15]	England	National Cancer Registration and Analysis Service	2001–2013	Calendar time
Chen et al. [65]	Taiwan	Longitudinal Health Insurance Database	1997–2011	Overall, other population characteristics
Stoyanov et al. [19]	Bulgaria	15 villages in the Vratza district	1965–1974	Sex, age
Visser et al. [14]	Europe	RARECARE database	1995–2002	Sex, age, geographical region
Wang et al. [20]	Taiwan	NHIR database	1997–2002	Overall, sex, age
Joung et al. [21]	South Korea	Korea National Cancer Incidence Database	1999–2012	Overall, sex, age
Janbabaei et al. [32]	Iran	Population-based cancer registry system of Mazandaran University of Medical Sciences	2014	Overall, sex
Sun et al. [54]	Taiwan	NHIR database	1997–2010	Overall, other
Cheon et al. [22]	South Korea	Pathologic records of 46 hospital and clinics	1985–1999	Overall, sex, age, calendar time
Chow et al. [23]	Sweden	Swedish Cancer Registry	1965–1983	Overall, sex, age, other
Balaji et al. [66]	USA	Memorial Sloan-Kettering Cancer Center	1989–1997	Overall
Kang et al. [67]	Taiwan	Tumour registry of Chang Gung Memorial Hospital	1992–2001	Overall
Holmäng et al. [39]	Sweden	Swedish Cancer Registry	1971–1998	Calendar time
Bermejo et al. [33]	Sweden	Swedish Cancer Registry	1961–2006	Sex
Hsiao et al. [24]	Taiwan	NHIR database	2000–2012	Overall, sex, age, geographical region, occupation, other
Raman et al. [40]	USA	SEER Registry	1973–2005	Overall, calendar time
Nakata et al. [25]	Japan	62 hospitals and clinics	1985–1994	Age, calendar time
Wihlborg and Johansen [41]	Denmark	Danish Cancer Registry	1944–2003	Calendar time
Wang et al. [42]	Taiwan	NHIR database	1997–2008	Calendar time
Shinka et al. [68]	Japan	Wakayama Medical College	1969–1984	Overall
Wright et al. [69]	USA	SEER Registry	1988–2003	Overall
Eylert et al. [16]	England	National Cancer Data Repository	1985–2009	Overall, calendar time
Antoni et al. [34]	Australia	Population-based cancer registries in five Australian states	1983–2007	Sex, calendar time, geographical region
Cauberg et al. [43]	Netherlands	Association of Comprehensive Cancer Centres population-based cancer registry	1995–2003	Calendar time
Woodford et al. [18]	Australia	Victorian Cancer Registry	2001–2011	Overall, age, calendar time
Mellemgaard et al. [28]	Denmark	Danish Cancer Registry	1943–1988	Age, calendar time
Yang et al. [70]	Taiwan	Taipei Veterans General Hospital	1983–1998	Overall
Huguet-Pérez et al. [71]	Spain	Fundació Puigvert	1980–1994	Overall
Munoz et al. [44]	USA	SEER Registry	1973–1976	Calendar time
Furukawa et al. [72]	Japan	Kobe University Graduate School of Medicine hospitals	1995–2003	Overall
Amar and Das [73]	USA	Kaiser Permanente Medical Center	-	Overall, other
Millán-Rodríguez et al. [74]	Spain	Fundació Puigvert	1968–1996	Overall, other
Hurle et al. [75]	Italy	United Hospitals of Bergamo	1986–1992	Overall, other
Schwartz et al. [76]	USA	Montefiore Medical Center	1972–1982	Overall, other
Pukkala et al. [48]	Denmark, Finland, Iceland, Norway, Sweden	Computerised population census in the countries listed	1943–1990	Occupation
Friis et al. [77]	Denmark	Prescription Database of North Jutland County; Danish Cancer Registry	1989–1995	Overall, other
McLaughlin et al. [50]	USA	Valley Forge satellite manufacturing complex in Pennsylvania	1962–2008	Occupation
Mok et al. [12]	South Korea	Korean Heart Study	1996–2012	Other
Christensen et al. [30]	Denmark	Danish Cancer Registry	1968–2012	Sex, other
Yang et al. [36]	USA	SEER Registry	1973–2010	Overall, race, other
Mathew et al. [37]	Worldwide	SEER Registry, Cancer Incidence in Five Continents	1973–1992	Race, geographical region
Lynch and Cohen [26]	USA	SEER Registry	1973–1987	Age, calendar time
McLaughlin et al. [50]	Sweden	Swedish Cancer-Environment Registry	1960–1979	Occupation
Devesa et al. [78]	USA	SEER Registry	1975–1985	Race
Lynge et al. [51]	Denmark	Danish Cancer Registry	1970–1987	Occupation
Chow et al. [38]	USA	SEER Registry	1975–1995	Race
Ericson et al. [53]	Sweden	Swedish Cancer Registry	1961–1999	Lynch syndrome
Premuzic et al. [29]	Croatia	University Hospital Centre Zagreb	2011–2016	Sex
Michalek et al. [27]	Denmark, Iceland, Finland, Norway and Sweden	Respective national cancer registries	1961–2005	Age, occupation, calendar time
Medunjanin et al. [31]	Croatia	Croatian National Cancer Registry	2001–2011	Sex, geographical region
Aragon-Ching et al. [79]	USA	SEER Registry	2000–2014	Overall
Wu et al. [42]	USA	SEER Registry	1988–2015	Calendar time
Fernandez Aparicio et al. [80]	Spain	31 hospitals in Spain	2017–2018	Overall
Tempo et al. [46]	Australia	South Australia Cancer Registry	1977–2013	Calendar time
Almås et al. [17]	Norway	Cancer Registry of Norway	1999–2018	Overall, age, calendar time
Michalek et al. [27]	Denmark, Iceland, Finland, Norway and Sweden	Respective national cancer registries	1961–2005	Occupation

The studies selected included data ranging from the years 1943 to 2018. Europe had the greatest number of studies (n = 26, 44%), followed by Asia (n = 15, 25%), North America (n = 14, 24%), Australia (three studies), and one worldwide study. Of the studies from Europe, 14 (54%) were conducted in the Nordic countries of Denmark, Finland, Iceland, Norway, or Sweden. Of the studies from Asia, seven (47%) were conducted in Taiwan. Nearly all studies employed a retrospective cohort study design except for two studies: Mok et al. and Aparicio et al., which were prospective cohort studies [12, 13]. Similarly, nearly all studies focused on the incidence of UTUC, with only one study by Visser and colleagues [14] reporting incidence together with prevalence.

The studies selected for this review included a wide variety of cancer registries and patient populations. Eleven studies (19%) used data from the SEER (Surveillance, Epidemiology, and End Results) registry in the USA. In total, 38 studies (64%) used International Classification of Diseases (ICD) codes to identify patients with a diagnosis of UTUC.

Seven corresponding authors were contacted for the raw data of graphs in their respective articles, to which four authors [15–18] replied with this data. In total, eight papers required data extraction using WebPlotDigitizer (Additional file 2).

### Incidence by age

In total, 12 studies reported incidence of UTUC according to age [14, 17–28]. The measures used to report incidence varied widely between different studies. For example, the study by Visser et al. used rate per million persons, whereas Cheon and colleagues used rate per 100,000 persons [14, 22]. Other measures used in other studies include incidence density per 10,000 person-years and standardised incidence ratios.

Overall, there appeared to be a higher incidence of UTUC with increasing age. This was seen in both males and females. Stoyanov and colleagues [19] reported an incidence of 11.22 per 100,000 persons in male 30–39 year olds, which increased to 17.30 per 100,000 persons in males aged 70 + . In females, the increase seen in age was higher, from 21.28 to 277.78 per 100,000 persons between 30 and 39 year olds and 70+, respectively. A similar increasing trend with age was reported by Visser et al. [14], with an incidence of 1.8 (standard error [SE] 0.1) per million persons in patients < 55 years old compared to 73.6 (SE 1.1) per million persons in 75 + year olds.

### Incidence by sex

The calculation of age-standardised rates (ASRs) varied according to what population was used in standardisation. In the 13 studies that reported on incidence of UTUC according to sex [14, 19–24, 29–34], the ASR values were adjusted based on different populations, including a European population [14], a standard world population [21], the World Health Organisation (WHO) standard population [32], the 1995 South Korean population [22], and population data from the Australian Bureau of Statistics [34].

The comparative incidence between males and females was not clear from the studies. Some studies reported a higher incidence in males than females. For example, Joung and colleagues [21] reported an ASR of 1.39 (males) vs. 0.49 (females) per 100,000 persons. Others such as Wang and colleagues [20] reported a higher incidence in females than males, with a standardised incidence ratio (SIR) of 15.2 (95% CI 12.7–17.9) for females vs. 8.1 (95% CI 6.5–10.2) for males.

### Incidence by race

Four studies compared incidence between different races [35–38]. The most comprehensive study was by Mathew and colleagues [37], who reported worldwide incidence of UTUC between 1973 and 1992. The study reported SIR values for, among others, Black Americans, White Americans, Jewish Israelis, and Chinese Singaporeans. The highest SIR for renal pelvis cancers in this study was seen in White Americans (0.79 per 100,000 person-years for males). Another study by Chow and colleagues [38] reported a lower ASR in Black American males (0.8 per 100,000 person-years) compared to White American males (1.5 per 100,000 person-years). This was similarly seen in Black American females (0.5 per 100,000 person-years) vs. White American females (0.7 per 100,000 person-years).

Another study by Li and colleagues, [36] which covered an even wider period (1973–2010) but focused on colorectal cancer survivors, reported a higher SIR of renal pelvis cancers in Black compared to White Americans (1.73 [95% CI 0.56–4.04] vs. 1.29 [0.97–1.69], respectively). The opposite was seen in ureter cancers, with White Americans having a higher SIR than Black Americans (1.49 [1.18–1.86] vs. 1.32 [0.16–4.78], respectively).

### Incidence by geographical region

Five studies reported on incidence of UTUC according to specific geographical regions [14, 24, 31, 34, 37]. The study by Mathew et al. [37] covered 10 regions for both males and females, and a further five regions for males. This study used data from the Cancer Incidence in Five Continents resource and found that the region with the highest SIR of renal pelvis cancers between 1973 and 1992 was the Bas-Rhin region in eastern France, with an SIR of 15.5 per 100,000 person-years in males.

Visser et al. [14] further reported incidence in different regions of Europe between 1995 and 2002 and found northern Europe to have the highest ASR of 12.8 (SE 0.3) per million persons. The high incidence of UTUC in regions with a high prevalence of Balkan endemic nephropathy (BEN) was studied by Medunjanin and colleagues [31], who reported a high incidence in Croatia geographically associated with a high prevalence of BEN, specifically in the Brod-Posavina county.

Differences between regions within countries were also studied. Hsiao et al. [24] studied UTUC incidence in Taiwan in patients who had undergone haemodialysis, finding the highest incidence in southern Taiwan for both patients who had haemodialysis and those who did not (38.6 and 1.35 per 10,000 person-years, respectively). Other regions in Taiwan followed a similar pattern, with northern Taiwan having the lowest incidence of UTUC in both groups of patients (11.3 and 0.33 per 10,000 person-years, respectively). In Australia, Antoni et al. [34] studied the incidence of renal pelvis cancer in five Australian states and reported the highest ASRs in New South Wales and Queensland.

### Incidence by calendar time

Nineteen studies compared UTUC incidence based on calendar time [15–18, 22, 25–28, 34, 39–47]. For three of these studies, more accurate numbers were obtained by contacting the study authors. The first of these studies was by Eylert and colleagues [16], who used data from the National Cancer Data Repository (NCDR) of England between 1985 and 2009 and found an overall increase throughout time in UTUC incidence for males, females, and both sexes combined. Similarly, Kockelbergh and colleagues [15] studied the National Cancer Registration and Analysis Service (NCRAS)—which contains data from the NCDR—from 2001 to 2013 and reported a similar overall increase in incidence throughout time. The third study with accurate numbers obtained from the study authors was by Woodford and colleagues [18], who studied the Victorian Cancer Registry of Australia from 2001 to 2011 and found the incidence of UTUC to be stable throughout time.

Other studies that showed a decreased incidence over time include the study by Antoni et al. [34], which reported a reduction in incidence in all five cancer registries in Australia that were studied from 1983–1987 to 2003–2007. The study by Wang et al. [42], which used data from the National Health Insurance Research Database in Taiwan and focused on patients with end-stage renal disease under maintenance dialysis, reported a reduced SIR_40-84_ of UTUC in males from 1998 (13.0 [95% CI 6.2–27.3]) to 2008 (9.5 [7.3–12.3]), but an increased SIR_40-84_ in females from 1998 (9.5 [7.3–12.3]) to 2008 (13.6 [11.4–16.4]).

### Incidence by occupation

Seven studies reported on UTUC incidence according to occupation [24, 27, 48–52]. Five of these studies were based on the national cancer registries and censuses of Nordic countries [27, 48, 50–52]. A total of 54 occupational categories are included in the comprehensive study by Michalek and colleagues [27] on five Nordic countries (Denmark, Iceland, Finland, Norway and Sweden) between 1961 and 2005. The occupation categories with the highest SIR were seamen (1.51 [95% CI 1.23–1.82]), printers (1.39 [1.11–1.71]), and welders (1.37 [1.03–1.78]). The study further classified occupations according to age at diagnosis, and the highest SIR was observed in seamen aged 30–49 years old (1.75 [0.96–2.94]). Michalek et al. published another study [52] using smoking prevalence data to adjust for smoking in males when reporting UTUC incidence. This study found the highest statistically significant smoking-adjusted SIR to be in physicians (1.63 [1.16–2.23]).

The study by Pukkala and colleagues [48] on the same five Nordic countries between 1943–1990 reported a similarly high SIR in seamen of 1.52 (1.26–1.85), while the highest SIR in women was seen in clerical workers (1.19 [1.08–1.31]). Moreover, McLaughlin et al. [50] reported SIR of renal pelvis cancers in 34 different occupations between 1960 and 1979 in Sweden, with the highest incidence found to be in judges (9.16), engineers and technicians in mining and metallurgy (3.29), and plumbers (2.17).

### Incidence by Lynch syndrome

Our search of the literature found only one epidemiological study on the incidence of UTUC in patients with Lynch syndrome. Many studies were excluded that focused instead on the incidence of Lynch syndrome in patients with UTUC, with other studies investigating the risks and survival rates of these patients.

The study by Ericson and colleagues [53] set out to assess cancer risk in patients whose parents developed any tumour associated with Lynch syndrome. The study used data from the Swedish Cancer Registry between 1961 and 1999. Patients were categorised according to familial risk groups, as well as the type of malignancy present in the parent. The overall SIR of UTUC in all patients was 1.2 (95% CI 0.9–1.5). The highest incidence of UTUC was seen in patients whose parent and sibling had an HNPCC-associated cancer, one of whom was under 50 years old when diagnosed—the SIR in this group was 29.6 (8.1–75.9).

### Incidence by other population characteristics

A number of studies included in this review reported an increased UTUC incidence in different populations and patient groups. For example, Sun et al. [54] studied patients with a recent urinary tract infection (UTI) diagnosis and found a higher incidence of UTUC in patients who had an upper UTI (1.38 per 10,000 person-years) compared to those who had a lower UTI (1.12) and no UTI (0.27). Another study by Chow et al. [23] to assess whether UTUC is linked to kidney and ureter stones reported a higher SIR of UTUC in patients who had a UTI at their index visit (6.7 [95% CI 2.4–14.5]) compared to those who did not (2.2 [1.6–3.0]).

### Prospective cohort studies

There were only two prospective cohort studies included in this review. Mok et al. [12] reported a higher incidence of ureter cancer in patients with a low estimated glomerular filtration rate (eGFR) of < 45 (13.7 per 10,000 person-years) compared to patients with a higher eGFR of ≥ 90 (0.3). The second study by Aparicio et al. [13] was a multicentre study of 31 institutions in Spain utilising a centralised database to report cases of UTUC prospectively. This study reported a relatively high incidence rate of 3.27 (95% CI 2.93–3.61) per 100,000 person-years, which the authors conclude is due to the prospective nature of their study.

### Prevalence

The only study to report on prevalence values for UTUC included in this review was by Visser et al. [14], which used the RARECARE database of rare cancers in Europe. However, estimated complete prevalence was only available for the index year 2003, and data was only used from 22 registries in 12 countries as representative of the EU27 countries. In total, the estimated complete prevalence for epithelial tumours of the renal pelvis and ureter was 101.0 (SE 1.5) per million persons. This was nearly double that of epithelial tumours of the penis, which was the next most prevalent cancer at 55.4 (SE 1.1) per million persons. The most common subtype of UTUC was transitional cell carcinoma at 93.2 (SE 1.4) per million persons, followed by adenocarcinoma at 1.4 (SE 0.2) per million persons, then squamous cell carcinoma (prevalence not reported).

## Discussion

Data from the 59 papers included in this review highlight a number of trends in the epidemiology of UTUC. Overall, there is an increase in incidence with age and specific occupations such as seamen and printers. The trend in incidence seen in other factors—sex, race, geographical region, and calendar time—was less clear. Single studies reported a high incidence of UTUC in patients with a history of an upper UTI, and patients with a low eGFR. Prevalence of UTUC was only reported by one study, which showed UTUC to be the most common of the rare urogenital cancers. Similarly, only one study reported the incidence of UTUC in patients with Lynch syndrome, and although this was limited to data obtained only from the Swedish Cancer Registry, it included data from a period of 40 years.

The general increase in UTUC incidence across time reported by some studies can be attributed to the introduction of computed tomography (CT) urography in the detection of UTUC [15]. CT urography is a recent introduction in the diagnosis of UTUC, which for many decades was dominated by intravenous urography. Several guidelines have only recently changed to reflect the higher diagnostic accuracy provided by CT urography, which is now the first-line imaging modality for investigating UTUC [55].

The relatively high incidence of UTUC reported in seamen has been proposed to be due to increased exposure to asbestos fibres in ships [27]. The association between occupational exposure and other urological cancers has been studied, with a high incidence of bladder cancer in seamen reported in a study on the Nordic Occupational Cancer cohort [56]. Exposure to other chemicals, such as the azo dyes used in the printing and dyeing industry, have also been linked to increased bladder cancer incidence [57]. This could explain the increased UTUC incidence observed in printers in the study reported by Michalek et al. [27]. A further aspect of occupational exposure was proposed by Michalek et al. [52] in their study of smoking-adjusted incidence, with the suggestion that exposure to phenacetin and X-rays/gamma rays could be the cause of a statistically significant high smoking-adjusted SIR in physicians, although this was also suggested to be due to surveillance bias and increased awareness of urothelial cancer symptoms by physicians.

The studies that reported values on race generally found a mixed picture in the incidence of UTUC between Black and White Americans. Mathew et al. and Chow et al. reported a higher SIR for renal pelvis cancers in White Americans compared to Black Americans [37, 38]. Racial disparities between Black and White Americans have been studied and have been found to exist in other urological cancers [58, 59]. The reason for this disparity is difficult to quantify, as it could be attributed to genetics, lifestyle factors such as smoking, or poorer access to healthcare.

Studies based in Taiwan report a relatively high incidence of UTUC due to the widespread consumption of aristolochic acid, an established risk factor for UTUC. This carcinogenic and nephrotoxic agent, which was banned in Taiwan in 2003, is present in herbal medicines and has been linked to an increased risk of urinary tract cancers [60]. Exploring the effect of the ban of aristolochic acid on UTUC incidence, Wang et al. [42] reported a decrease in the SIR_40-84_ in both males and females after the introduction of the ban, although the authors note that there remains another carcinogenic and nephrotoxic agent (Xi-Xin) similar to aristolochic acid in circulation in Taiwan that requires further study.

The SEER (Surveillance, Epidemiology, and End Results) Program is a database that collects data on cancer incidence in the USA and is a common tool used in epidemiological studies of cancer [61]. However, many excluded studies from this review considered the SEER category of ‘kidney and renal pelvis’ cancers as cancers of the renal pelvis. Kidney and renal pelvis cancers are not alike as they originate from different tissues and are histologically different, namely, renal cell carcinoma and urothelial carcinoma. This is reflected in the different ICD-10 codes assigned to each cancer: C64 (Malignant neoplasm of kidney, except renal pelvis), C65 (Malignant neoplasm of renal pelvis), and C66 (Malignant neoplasm of ureter) [62].

Similarly, many of the studies excluded from this review cite the comprehensive study by Siegel and colleagues [63], but this paper has the same limitation as above in that renal pelvis cancers are grouped together with kidney cancers. Moreover, studies that mention a high incidence of UTUC in endemic areas of blackfoot disease do not report UTUC separately, instead grouping UTUC with other urinary cancers. Future studies reporting on renal pelvis cancers should ensure that there is a clear delineation between these two types of cancers with a different aetiology and histology.

Full-text screening identified only one study [14] reporting on the prevalence of UTUC that met our inclusion criteria. While data on the incidence of UTUC is useful in identifying trends, the lack of prevalence studies on UTUC needs to be addressed in order to provide a better picture of the burden of UTUC in different populations.

A notable limitation that was identified through the use of the JBI checklist was that many studies did not report the 95% confidence intervals of their incidence values. Furthermore, the JBI checklist also highlighted that many papers in our review did not mention ethical approval, which should be required even if the study design is observational, as databases with personally identifiable information may be used during the study. Future epidemiological studies should ensure that appropriate ethical approval is obtained before commencing the study.

A limitation of this review is that the data from the studies selected was found to be heterogeneous, and hence a qualitative synthesis of the data was done in place of a meta-analysis to reflect the nature of the data. Future studies on the epidemiology of UTUC should consider reporting incidence data using similar metrics (such as those for age standardisation) in order to allow for a more accurate quantitative comparison.

## Conclusion

Although UTUC is a relatively rare urological cancer, it is still an important public health concern in many areas and patient populations around the world. The present review highlights an increased incidence of UTUC seen in certain groups, including increasing age and certain occupations such as seamen. UTUC is more endemic in certain geographical regions of the world where there are associated risk factors. The trend of UTUC incidence for sex, race, and calendar time is less clear due to a wide variety of measures used to report incidence. There is also sparse literature on the prevalence of UTUC, and further studies using this important epidemiological metric are needed to know the true disease burden of UTUC. The results of this review provide epidemiologists, public health specialists, and clinicians a better understanding of the epidemiology of UTUC in order to guide diagnosis and prevention.

## Supplementary Information

**Additional file 1**. Search strategy.

**Additional file 2**. Study descriptives sorted by population characteristics.

## Data Availability

The datasets used and analysed during the current study are available from the corresponding author on reasonable request.

## Abbreviations

UTUC: Upper urinary tract urothelial carcinoma
JBI: Joanna Briggs Institute
SEER: Surveillance, Epidemiology, and End Results
CI: Confidence interval
ASR: Age-standardised rate
SIR: Standardised incidence ratio
SE: Standard error
UTI: Urinary tract infection
