# Supplementary material for: The incidence and prevalence of upper tract urothelial carcinoma: a systematic review
Source: BMC Urol. 2021 Aug 17;21:110. doi: 10.1186/s12894-021-00876-7 (PMC8369798; doi:10.1186/s12894-021-00876-7)
Supplement: Supplementary file 1 — Additional file 1. Search strategy. [file 12894_2021_876_MOESM1_ESM.docx]

**Additional file 1.** Search strategy

- **MEDLINE**

Upper urinary tract urothelial carcinoma syntax:

"Ureteral neoplasms"[MeSH] OR "Ureteral neoplasms"[TIAB] OR "Ureter tumor*"[TIAB] OR "Ureter tumour*"[TIAB] OR "Ureteral tumor*"[TIAB] OR "Ureteral tumour*"[TIAB]

OR "Upper urinary tract urothelial carcinoma*"[TIAB] OR "Upper tract urothelial carcinoma*"[TIAB] OR "Upper urinary tract urothelial cell carcinoma*"[TIAB] OR "Upper tract urothelial cell carcinoma*"[TIAB] OR "Upper urinary tract transitional cell carcinoma*"[TIAB] OR "Upper tract transitional cell carcinoma*"[TIAB] OR "UTUC"[TIAB] OR "Upper urinary tract cancer*"[TIAB] OR "Upper urinary tract neoplasm*"[TIAB] OR "Upper urinary tract tumor*"[TIAB] OR "Upper urinary tract tumour*"[TIAB] OR "Upper urinary tract malignanc*"[TIAB]

OR "Renal pelvis urothelial carcinoma*"[TIAB] OR "Renal pelvis urothelial cell carcinoma*"[TIAB] OR "Renal pelvis transitional cell carcinoma*"[TIAB] OR "Renal pelvis neoplasm*"[TIAB] OR "Renal pelvis cancer*"[TIAB] OR "Renal pelvis tumor*"[TIAB] OR "Renal pelvis tumour*"[TIAB] OR "Renal pelvis malignanc*"[TIAB]

OR "Kidney pelvis urothelial carcinoma*"[TIAB] OR "Kidney pelvis urothelial cell carcinoma*"[TIAB] OR "Kidney pelvis transitional cell carcinoma*"[TIAB] OR "Kidney pelvis neoplasm*"[TIAB] OR "Kidney pelvis cancer*"[TIAB] OR "Kidney pelvis tumor*"[TIAB] OR "Kidney pelvis tumour*"[TIAB] OR "Kidney pelvis malignanc*"[TIAB]

OR "Pyelocaliceal urothelial carcinoma*"[TIAB] OR "Pyelocaliceal urothelial cell carcinoma*"[TIAB] OR "Pyelocaliceal transitional cell carcinoma*"[TIAB] OR "Pyelocaliceal neoplasm*"[TIAB] OR "Pyelocaliceal cancer*"[TIAB] OR "Pyelocaliceal tumor*"[TIAB] OR "Pyelocaliceal tumour*"[TIAB] OR "Pyelocaliceal malignanc*"[TIAB]

OR "Pyelocalyceal urothelial carcinoma*"[TIAB] OR "Pyelocalyceal urothelial cell carcinoma*"[TIAB] OR "Pyelocalyceal transitional cell carcinoma*"[TIAB] OR "Pyelocalyceal neoplasm*"[TIAB] OR "Pyelocalyceal cancer*"[TIAB] OR "Pyelocalyceal tumor*"[TIAB] OR "Pyelocalyceal tumour*"[TIAB] OR "Pyelocalyceal malignanc*"[TIAB]

OR “Colorectal Neoplasms, Hereditary Nonpolyposis”[MeSH] OR “Colorectal Neoplasms, Hereditary Nonpolyposis”[TIAB] OR “Lynch syndrome*”[TIAB] OR “HNPCC”[TIAB]

Epidemiology syntax:

"Incidence"[MeSH] OR "Prevalence"[MeSH] OR "Epidemiology"[MeSH] OR “Incidence*”[TIAB] OR “Prevalence*”[TIAB] OR “Epidem*”[TIAB]

- **Embase**

Upper urinary tract urothelial carcinoma syntax:

‘Ureter tumor’/exp OR ‘Ureter tumor*’:ti,ab OR ‘Ureter tumour*’:ti,ab OR ‘Ureteral tumor*’:ti,ab OR ‘Ureteral tumour*’:ti,ab OR ‘Ureteral neoplasm’:ti,ab

OR ‘Upper urinary tract urothelial carcinoma*’:ti,ab OR ‘Upper tract urothelial carcinoma*’:ti,ab OR ‘Upper urinary tract urothelial cell carcinoma*’:ti,ab OR ‘Upper tract urothelial cell carcinoma*’:ti,ab OR ‘Upper urinary tract transitional cell carcinoma*’:ti,ab OR ‘Upper tract transitional cell carcinoma*’/exp OR ‘UTUC’:ti,ab OR ‘Upper urinary tract cancer*’:ti,ab OR ‘Upper urinary tract neoplasm*’:ti,ab OR ‘Upper urinary tract tumor*’:ti,ab OR ‘Upper urinary tract tumour*’:ti,ab OR ‘Upper urinary tract malignanc*’:ti,ab

OR ‘Renal pelvis urothelial carcinoma*’:ti,ab OR ‘Renal pelvis urothelial cell carcinoma*’:ti,ab OR ‘Renal pelvis transitional cell carcinoma*’:ti,ab OR ‘Renal pelvis neoplasm*’:ti,ab OR ‘Renal pelvis cancer*’:ti,ab OR ‘Renal pelvis tumor*’:ti,ab OR ‘Renal pelvis tumour*’:ti,ab OR ‘Renal pelvis malignanc*’:ti,ab

OR ‘Kidney pelvis carcinoma*’/exp OR ‘Kidney pelvis cancer*’/exp OR ‘Kidney pelvis tumor*’/exp OR ‘Kidney pelvis carcinoma*’:ti,ab OR ‘Kidney pelvis cancer*’:ti,ab OR ‘Kidney pelvis tumor*’:ti,ab OR ‘Kidney pelvis urothelial carcinoma*’:ti,ab OR ‘Kidney pelvis urothelial cell carcinoma*’:ti,ab OR ‘Kidney pelvis transitional cell carcinoma*’:ti,ab OR ‘Kidney pelvis neoplasm*’:ti,ab OR ‘Kidney pelvis cancer*’:ti,ab OR ‘Kidney pelvis tumor*’:ti,ab OR ‘Kidney pelvis tumour*’:ti,ab OR ‘Kidney pelvis malignanc*’:ti,ab

OR ‘Pyelocaliceal urothelial carcinoma*’:ti,ab OR ‘Pyelocaliceal urothelial cell carcinoma*’:ti,ab OR ‘Pyelocaliceal transitional cell carcinoma*’:ti,ab OR ‘Pyelocaliceal neoplasm*’:ti,ab OR ‘Pyelocaliceal cancer*’:ti,ab OR ‘Pyelocaliceal tumor*’:ti,ab OR ‘Pyelocaliceal tumour*’:ti,ab OR ‘Pyelocaliceal malignanc*’:ti,ab

OR ‘Pyelocalyceal urothelial carcinoma*’:ti,ab OR ‘Pyelocalyceal urothelial cell carcinoma*’:ti,ab OR ‘Pyelocalyceal transitional cell carcinoma*’:ti,ab OR ‘Pyelocalyceal neoplasm*’:ti,ab OR ‘Pyelocalyceal cancer*’:ti,ab OR ‘Pyelocalyceal tumor*’:ti,ab OR ‘Pyelocalyceal tumour*’:ti,ab OR ‘Pyelocalyceal malignanc*’:ti,ab

OR ‘Hereditary nonpolyposis colorectal cancer’/exp OR ‘Hereditary nonpolyposis colorectal cancer’:ti,ab OR ‘Lynch syndrome*’:ti,ab ‘Colorectal Neoplasms, Hereditary Nonpolyposis’:ti,ab OR ‘HNPCC’:ti,ab

Epidemiology syntax:

‘Incidence*’/exp OR ‘Cancer incidence*’/exp OR ‘Prevalence*’/exp OR ‘Epidemiolog*’/exp OR ‘Cancer epidemiolog*’/exp OR ‘Incidence*’:ti,ab OR ‘Cancer incidence*’:ti,ab OR ‘Prevalence*’:ti,ab OR ‘Epidemiolog*’:ti,ab OR ‘Cancer epidemiolog*’:ti,ab

- **Web of Science**

Upper urinary tract urothelial carcinoma syntax:

TS=(“Ureteral neoplasm*”) OR TS=(“Ureter tumor*”) OR TS=(“Ureter tumour*”) OR TS=(“Ureteral tumor*”) OR TS=(“Ureteral tumour*”)

OR TS=(“Upper urinary tract urothelial carcinoma*”) OR TS=(“Upper tract urothelial carcinoma*”) OR TS=(“Upper urinary tract urothelial cell carcinoma*”) OR TS=(“Upper tract urothelial cell carcinoma*”) OR TS=(“Upper urinary tract transitional cell carcinoma*”) OR TS=(“Upper tract transitional cell carcinoma*”) OR TS=(“UTUC) OR TS=(“Upper urinary tract cancer*”) OR TS=(“Upper urinary tract neoplasm*”) OR TS=(“Upper urinary tract tumor*”) OR TS=(“Upper urinary tract tumour*”) OR TS=(“Upper urinary tract malignanc*”)

OR TS=(“Renal pelvis urothelial carcinoma*”) OR TS=(“Renal pelvis urothelial cell carcinoma*”) OR TS=(“Renal pelvis transitional cell carcinoma*”) OR TS=(“Renal pelvis neoplasm*”) OR TS=(“Renal pelvis cancer*”) OR TS=(“Renal pelvis tumor*”) OR TS=(“Renal pelvis tumour*”) OR TS=(“Renal pelvis malignanc*”)

OR TS=(“Kidney pelvis urothelial carcinoma*”) OR TS=(“Kidney pelvis urothelial cell carcinoma*”) OR TS=(“Kidney pelvis transitional cell carcinoma*”) OR TS=(“Kidney pelvis neoplasm*”) OR TS=(“Kidney pelvis cancer*”) OR TS=(“Kidney pelvis tumor*”) OR TS=(“Kidney pelvis tumour*”) OR TS=(“Kidney pelvis malignanc*”)

OR TS=(“Pyelocaliceal urothelial carcinoma*”) OR TS=(“Pyelocaliceal urothelial cell carcinoma*”) OR TS=(“Pyelocaliceal transitional cell carcinoma*”) OR TS=(“Pyelocaliceal neoplasm*”) OR TS=(“Pyelocaliceal cancer*”) OR TS=(“Pyelocaliceal tumor*”) OR TS=(“Pyelocaliceal tumour*”) OR TS=(“Pyelocaliceal malignanc*”)

OR TS=(“Pyelocalyceal urothelial carcinoma*”) OR TS=(“Pyelocalyceal urothelial cell carcinoma*”) OR TS=(“Pyelocalyceal transitional cell carcinoma*”) OR TS=(“Pyelocalyceal neoplasm*”) OR TS=(“Pyelocalyceal cancer*”) OR TS=(“Pyelocalyceal tumor*”) OR TS=(“Pyelocalyceal tumour*”) OR TS=(“Pyelocalyceal malignanc*”)

OR TS=(“Colorectal Neoplasms, Hereditary Nonpolyposis”) OR TS=(“Lynch syndrome*”) OR TS=(“HNPCC”)

Epidemiology syntax:

TS=(“Incidence*”) OR TS=(“Prevalence*”) OR TS=(“Epidemiolog*”)
