# Supplementary material for: The incidence and prevalence of upper tract urothelial carcinoma: a systematic review
Source: BMC Urol. 2021 Aug 17;21:110. doi: 10.1186/s12894-021-00876-7 (PMC8369798; doi:10.1186/s12894-021-00876-7)
Supplement: Supplementary file 2 — Additional file 2. Study descriptives sorted by population characteristics. [file 12894_2021_876_MOESM2_ESM.docx]

**Additional file 2.** Study descriptives sorted by population characteristics

# Studies by age

| Study | Values reported |
| --- | --- |
| Stoyanov et al. 1978 | Incidence rate per 100,000 persons:  Male  **All ages**: 43.25  **30-39**: 11.22  **40-49**: 59.85  **50-59**: 131.17  **60-69**: 87.52  **70+**: 17.30  Female  **All ages**: 85.91  **30-39**: 21.28  **40-49**: 38.81  **50-59**: 186.44  **60-69**: 167.57  **70+**: 277.78 |
| Visser et al. 2012 | Rate per million (SE):  **<55**: 1.8 (0.1)  **55-64**: 26.5 (0.6)  **65-74**: 60.3 (0.9)  **75+**: 73.6 (1.1) |
| Wang et al. 2015 | SIR (95% CI):  Male  **<40**: 65.3 (32.7-130.7)  **40-49**: 10.1 (5.6-18.2)  **50-59**: 5.0 (3.2-7.7)  **60-69**: 2.3 (1.6-3.4)  **>70**: 0.7 (0.4-1.4)  Female  **<40**: 35.5 (11.1-107.1)  **40-49**: 22.7 (14.9-34.5)  **50-59**: 10.7 (7.6-14.9)  **60-69**: 3.4 (2.5-4.6)  **>70**: 2.3 (1.6-3.2) |
| Joung et al. 2017 | ASR (per 100,000 persons):  **<40y**: 0.01  **40-59**: 0.19  **60-69**: 0.33  **≥70**: 0.34 |
| Cheon et al. 2002 | ASR (per 100,000 persons):  Male  **20-29**: 0.05  **30-39**: 0.59  **40-49**: 1.59  **50-59**: 9.27  **60-69**: 17.10  **70+**: 25.40  Female  **20-29**: 0.05  **30-39**: 0.09  **40-49**: 0.54  **50-59**: 1.91  **60-69**: 5.38  **70+**: 5.15  Total (male & female)  **20-29**: 0.05  **30-39**: 0.35  **40-49**: 1.07  **50-59**: 5.55  **60-69**: 10.61  **70+**: 12.09 |
| Chow et al. 1997 | SIR (95% CI):  **Age entered cohort**  **<30**: 0.0 (0.0-31.5)  **30-39**: 2.9 (0.3-10.3)  **40-49**: 2.5 (1.0-5.2)  **50-59**: 2.5 (1.4-4.1)  **60-69**: 2.1 (1.1-3.6)  **70+**: 3.1 (1.4-5.9) |
| Hsiao et al. 2016 | Incidence density (per 10,000 person-years):  No hemodialysis  **20-59**: 0.19  **60-74**: 0.65  **≥75**: 1.64  Yes hemodialysis  **20-59**: 18.7  **60-74**: 30.5  **≥75**: 12.6 |
| Nakata et al. 1998 | ASR per 100,000 persons: (extracted using WebPlotDigitizer)  Males  **40**: 0.37  **50**: 1.36  **60**: 3.85  **70**: 9.59  **80**: 10.84  **90**: 8.0 |
| Woodford et al. 2016 | ASR:  Males  **<50**: 0.0966  **50-59**: 1.17074  **60-69**: 4.25346  **70-79**: 12.6867  **80+**: 15.4649  Females  **<50**: 0.0155  **50-59**: 0.62635  **60-69**: 2.19992  **70-79**: 7.42519  **80+**: 7.97457 |
| Mellemgaard et al. 1993 | Incidence rate per 100,000 person-years: (extracted using WebPlotDigitizer)  Female, 1963-1967  **45**: 0.34  **50**: 0.66  **55**: 1.12  **60**: 1.74  **65**: 2.53  **70**: 3.22  **75**: 3.58  **80**: 3.42  **85**: 3.19 |
| Lynch and Cohen 1995 | ASR per 100,000 persons (ureter): (extracted using WebPlotDigitizer)  Male  **Age at diagnosis**  **40-44**: 0  **45-49**: 0.22  **50-54**: 0.58  **55-59**: 1.38  **60-64**: 2.17  **65-69**: 3.6  **70-74**: 5.95  **75-79**: 6.86  **80-84**: 9.5  **85+**: 3.21  Female  **Age at diagnosis**  **40-44**: 0  **45-49**: 0  **50-54**: 0  **55-59**: 0.48  **60-64**: 0.89  **65-69**: 1.55  **70-74**: 2.61  **75-79**: 2.73  **80-84**: 3.3  **85+**: 2.53 |
| Michalek et al. 2019 | SIR (95% CI):  **Age at diagnosis**  **30-49 years old**  Clerical workers: 0.97 (0.75-1.23)  Electrical workers: 0.98 (0.63-1.46)  Food workers: 1.18 (0.68-1.92)  Forestry workers: 1.13 (0.69-1.75)  Gardeners: 0.62 (0.23-1.34)  Packers: 1.72 (1.12-2.52)  Painters: 1.30 (0.67-2.28)  Printers: 0.49 (0.13-1.27)  Public safety workers: 0.76 (0.33-1.50)  Seamen: 1.75 (0.96-2.94)  Textile workers: 0.89 (0.43-1.64)  Transport workers: 0.87 (0.42-1.61)  Welders: 1.54 (0.80-2.70)  Woodworkers: 0.89 (0.59-1.28)  **50-69 years old**  Clerical workers: 1.17 (1.08-1.26)  Electrical workers: 1.15 (1.00-1.32)  Food workers: 1.30 (1.14-1.47)  Forestry workers: 0.73 (0.62-0.84)  Gardeners: 0.36 (0.26-0.50)  Packers: 1.27 (1.10-1.45)  Painters: 1.35 (1.13-1.61)  Printers: 1.35 (1.09-1.66)  Public safety workers: 1.38 (1.14-1.64)  Seamen: 1.57 (1.30-1.87)  Textile workers: 1.22 (1.06-1.40)  Transport workers: 0.93 (0.76-1.12)  Welders: 1.31 (1.00-1.68)  Woodworkers: 0.74 (0.65-0.83)  **≥70 years old**  Clerical workers: 1.22 (1.12-1.32)  Electrical workers: 1.26 (1.07-1.48)  Food workers: 1.01 (0.86-1.18)  Forestry workers: 0.69 (0.60-0.80)  Gardeners: 0.56 (0.43-0.72)  Packers: 1.14 (0.98-1.32)  Painters: 1.04 (0.83-1.29)  Printers: 1.58 (1.25-1.96)  Public safety workers: 1.40 (1.15-1.70)  Seamen: 1.38 (1.09-1.73)  Textile workers: 1.24 (1.09-1.42)  Transport workers: 1.55 (1.32-1.81)  Welders: 1.42 (1.02-1.94)  Woodworkers: 0.87 (0.78-0.98) |
| Almås et al. 2021 | ASR (per 100,000 persons):  **Age**  **0-19**: 0  **20-29**: 0.03  **30-39**: 0.05  **40-49**: 0.5  **50-59**: 2.25  **60-69**: 8.04  **70-79**: 18.54  **80+**: 18.81 |

# Studies by sex

| Study | Values reported |
| --- | --- |
| Stoyanov et al. 1978 | Incidence rate per 100,000 persons:  **Male**  All ages: 43.25  **Female**  All ages: 85.91 |
| Visser et al. 2012 | ASR per million persons (SE):  **Male**: 16.5 (0.2)  **Female**: 7.3 (0.1) |
| Wang et al. 2015 | SIR:  **Male**: 8.1 (6.5-10.2)  **Female**: 15.2 (12.7-17.9) |
| Joung et al. 2017 | ASR (per 100,000 persons):  **Male**: 1.39  **Female**: 0.49 |
| Janbabaei et al. 2018 | SIR (per 100,000 persons):  Ureter  **Male**: 0.05  **Female**: 0.09 |
| Cheon et al. 2002 | ASR (per 100,000 persons):  **Male**  Total (all ages): 2.95  **Female**  Total: 0.97 |
| Chow et al. 1997 | SIR (95% CI):  **Male**: 1.9 (1.3-2.7)  **Female**: 5.0 (2.9-8.0) |
| Bermejo et al. 2009 | SIR (95% CI):  Renal pelvis  **Female**: 40.1 (30.0-53.5)  **Male**: 34.5 (29.2-40.7) |
| Hsiao et al. 2016 | Incidence density (per 10,000 person-years):  No hemodialysis  **Female**: 0.58  **Male**: 0.72  Yes hemodialysis  **Female**: 25.9  **Male**: 18.1 |
| Antoni et al. 2014 | ASR (for renal pelvis cancer):  **Men**, 1983-1987  All 5 registries: 2.02  **Men**, 2003-2007  All 5 registries: 1.24  **Women**, 1983-1987  All 5 registries: 2.26  **Women**, 2003-2007  All 5 registries: 1.08 |
| Christensen et al. 2017 | SIR (renal pelvis and ureter) (95% CI):  **Men**: 0.78 (0.56-1.05)  **Women**: 0.41 (0.05-1.48) |
| Premuzic et al. 2017 | SIR (for ureter):  **Female**: 11.1 (10.8-11.4) |
| Medunjanin et al. 2020 | SIR (95% CI):  Brod-Posavina  **Male**: 3.03 (2.04-4.01)  **Female**: 4.96 (3.59-6.34)  Zagreb  **Male**: 1.00 (0.74-1.25)  **Female**: 0.98 (0.70-1.26) |

# Studies by race

| Study | Values reported |
| --- | --- |
| Noone et al. 2017 | Incidence rate (renal pelvis):  **White**: 0.7  **Black**: 0.6  **API (Asian/Pacific Islander)**: 0.6 |
| Yang et al. 2017 | SIR:  Renal pelvis  **White**: 1.29 (0.97–1.69)  **Black**: 1.73 (0.56–4.04)  **Other**: 1.66 (0.72–3.27)  **Unknown**: 0 (0–16.44)  Ureter  **White**: 1.49 (1.18–1.86)  **Black**: 1.32 (0.16–4.78)  **Other**: 1.68 (0.68–3.46)  **Unknown**: 0 (0–25.38) |
| Mathew et al. 2002 | SIR per 100,000 person-years (renal pelvis, 1988-1992): (extracted using WebPlotDigitizer)  Males  U.S., SEER, **Black**: 0.45  U.S., SEER, **White**: 0.79  U.S., L.A., **Hispanic**, **White**: 0.67  New Zealand, **Non-Maori**: 0.28  Israel, **all Jews**: 0.58  Singapore, **Chinese**: 0.19  Females  U.S., SEER, **Black**: 0.11  U.S., SEER, **White**: 0.34  U.S., L.A., **Hispanic**, **White**: 0.34  New Zealand, **Non-Maori**: 0.12  Israel, **all** **Jews**: 0.04 |
| Chow et al. 1999 | ASR (per 100,000 person-years):  **White**  Men: 1.5  Women: 0.7  **Black**  Men: 0.8  Women: 0.5 |

# Studies by geographical region

| Study | Values reported |
| --- | --- |
| Visser et al. 2012 | ASR per million persons (SE):  **Northern Europe**: 12.8 (0.3)  **Central Europe**: 12.7 (0.2)  **Eastern Europe**: 8.4 (0.3)  **Southern Europe**: 11.0 (0.2)  **UK and Ireland**: 10.8 (0.2) |
| Hsiao et al. 2016 | Incidence density (per 10,000 person-years):  No hemodialysis  **North**: 0.33  **Central**: 0.72  **South**: 1.35  **East and Island**: -  Yes hemodialysis  **North**: 11.3  **Central**: 28.3  **South**: 38.6  **East and Island**: 4.4 |
| Antoni et al. 2014 | ASR (for renal pelvis cancer)  Men, 1983-1987  **NSW**: 2.00  **QL**: 2.52  **SA**: 1.94  **Victoria**: 1.82  **WA**: 1.84  Men, 2003-2007  **NSW**: 1.40  **QL**: 1.19  **SA**: 1.45  **Victoria**: 1.02  **WA**: 1.16  Women, 1983-1987  **NSW**: 3.13  **QL**: 3.22  **SA**: 1.59  **Victoria**: 1.07  **WA**: 1.16  Women, 2003-2007  **NSW**: 1.28  **QL**: 1.43  **SA**: 0.84  **Victoria**: 0.70  **WA**: 0.88 |
| Mathew et al. 2002 | SIR per 100,000 person-years (renal pelvis, 1988-1992): (extracted using WebPlotDigitizer)  Males  **Canada, Alberta**: 0.57  **Canada, British Columbia**: 0.45  **Sweden**: 0.79  **Denmark**: 0.90  **France, Bas-Rhin**: 0.58  **Germany, Eastern States**: 0.51  **Italy, Varese**: 0.86  **Switzerland, Geneva**: 0.94  **U.K., South Thames**: 0.12  **Australia, N.S.W.:** 0.82  **Japan, Miyagi**: 0.66  **China, Shanghai**: 0.19  Females  **Canada, Alberta**: 0.17  **Canada, British Columbia**: 0.25  **Sweden**: 0.51  **Denmark**: 0.67  **Germany, Eastern States**: 0.31  **Italy, Varese**: 0.35  **Switzerland, Geneva**: 0.39  **Australia, N.S.W.**: 1.29 |
| Medunjanin et al. 2019 | SIR (95% CI):  **County**  **Brod-Posavina**: 3.90 (3.08-4.72)  **Vukovar-Srijem**: 2.10 (1.53-2.67)  **Dubrovnik-Neretva**: 1.27 (0.74-1.80)  **Split-Dalmatia**: 1.11 (0.85-1.37)  **Karlovac**: 1.10 (0.65-1.54)  **Osijek-Baranja**: 1.07 (0.76-1.38)  **City of Zagreb**: 0.98 (0.80-1.17)  **Virovitica-Podravina**: 0.93 (0.38-1.48)  **Primorje-Gorski Kotar**: 0.83 (0.57-1.10)  **Istria**: 0.82 (0.50-1.15)  **Sisak-Moslavina**: 0.82 (0.48-1.17)  **Pozega-Slavonia**: 0.81 (0.28-1.35)  **Zadar**: 0.80 (0.45-1.15)  **Medimurje**: 0.69 (0.26-1.12)  **Koprivnica-Krizevci**: 0.67 (0.27-1.07)  **Lika-Senj**: 0.64 (0.13-1.16)  **Bjelovar-Bilogora**: 0.63 (0.26-0.99)  **Sibenik-Knin**: 0.54 (0.21-0.88)  **Zagreb**: 0.39 (0.20-0.57)  **Varazdin**: 0.38 (0.13-0.62)  **Krapina-Zagorje**: 0.38 (0.10-0.65) |

# Studies by calendar time

| Study | Values reported |
| --- | --- |
| Chernozemsky et al. 1977 | ASR (per 100,000 persons):  **1965-1969**: 16.9  **1970-1974**: 22.8 |
| Kockelbergh et al. 2017 | ASR (95% CI):  Males  **2001**: 2.402422871 (2.170448883-2.634396858)  **2002**: 2.526594468 (2.289440452-2.763748485)  **2003**: 2.587593235 (2.352754722-2.822431749)  **2004**: 2.629075689 (2.392977629-2.865173749)  **2005**: 3.022334535 (2.769359166-3.275309905)  **2006**: 3.274716137 (3.010487372-3.538944903)  **2007**: 3.29503761 (3.035712828-3.554362391)  **2008**: 3.569719928 (3.298709841-3.840730015)  **2009**: 3.918199588 (3.638873151-4.197526024)  **2010**: 3.792088642 (3.521814848-4.062362436)  **2011**: 4.254586961 (3.969496213-4.539677709)  **2012**: 4.327812759 (4.044038579-4.61158694)  **2013**: 4.070431976 (3.796790336-4.344073617)  Females  **2001**: 1.118665373 (0.983257793-1.254072953)  **2002**: 1.080759999 (0.947080501-1.214439498)  **2003**: 1.253495715 (1.111383734-1.395607697)  **2004**: 1.229348333 (1.088849869-1.369846797)  **2005**: 1.353615405 (1.205222223-1.502008588)  **2006**: 1.483433101 (1.330118044-1.636748158)  **2007**: 1.539982159 (1.38646162-1.693502697)  **2008**: 1.643315855 (1.484370727-1.802260983)  **2009**: 1.810618127 (1.644352711-1.976883542)  **2010**: 1.795917293 (1.631109958-1.960724628)  **2011**: 2.070696491 (1.893983876-2.247409107)  **2012**: 2.132378914 (1.956269677-2.308488151)  **2013**: 1.895839207 (1.731358093-2.06032032) |
| Cheon et al. 2002 | Incidence rate (per 100,000 persons):  **1985-1989**: 0.20  **1990-1994**: 0.55  **1995-1999**: 1.25 |
| Holmäng et al. 2013 | Incidence (per 100,000 persons):  Male  **1971**: 2.3  **1974**: 1.5  **1977**: 3.2  **1980**: 3.4  **1983**: 3.7  **1986**: 4.6  **1989**: 3.3  **1992**: 3.0  **1995**: 3.2  **1998**: 3.8  Female  **1971**: 1.4  **1974**: 1.6  **1977**: 1.9  **1980**: 2.3  **1983**: 2.1  **1986**: 1.8  **1989**: 2.6  **1992**: 2.0  **1995**: 1.3  **1998**: 2.1 |
| Raman et al. 2011 | Incidence rates (per 100,000 person-years):  Total (ureter + renal pelvis)  **1973**: 1.88  **2005**: 2.06  Ureter  **1973**: 0.69  **2005**: 0.91  Renal pelvis  **1973**: 1.19  **2005**: 1.15 |
| Nakata et al. 1998 | ASR per 100,000 persons: (extracted using WebPlotDigitizer)    Male  **1985**: 1.58  **1986**: 1.65  **1987**: 2.15  **1988**: 2.14  **1989**: 1.43  **1990**: 1.11  **1991**: 1.11  **1992**: 1.63  **1993**: 1.54  **1994**: 1.32  Female  **1985**: 0.14  **1986**: 0.31  **1987**: 0.42  **1988**: 0.65  **1989**: 0.19  **1990**: 0.42  **1991**: 0.41  **1992**: 0.43  **1993**: 0.33  **1994**: 0.37 |
| Wihlborg et al. 2010 | ASR (per 100,000 person-years):  Men  **1944-1948**: 0.21  **1949-1953**: 0.25  **1954-1958**: 0.54  **1959-1963**: 0.75  **1964-1968**: 1.07  **1969-1973**: 1.86  **1974-1978**: 2.26  **1979-1983**: 2.46  **1984-1988**: 2.75  **1989-1993**: 2.73  **1994-1998**: 2.64  **1999-2003**: 2.45  Women  **1944-1948**: 0.4  **1949-1953**: 0.08  **1954-1958**: 0.31  **1959-1963**: 0.44  **1964-1968**: 0.56  **1969-1973**: 0.88  **1974-1978**: 1.30  **1979-1983**: 1.52  **1984-1988**: 1.84  **1989-1993**: 1.50  **1994-1998**: 1.41  **1999-2003**: 1.25 |
| Wang et al. 2014 | SIR_40−84_:  Male  **1998**: 13.0 (6.2–27.3)  **1999**: 13.9 (8.8–22.1)  **2000**: 12.2 (7.8–19.1)  **2001**: 12.9 (8.9–18.7)  **2002**: 11.9 (8.5–16.6)  **2003**: 13.9 (10.5–18.4)  **2004**: 13.9 (10.5–18.4)  **2005**: 7.2 (5.2–10.0)  **2006**: 9.4 (7.2–12.3)  **2007**: 9.5 (7.3–12.3)  **2008**: 9.5 (7.3–12.3)  Female  **1998**: 9.5 (7.3–12.3)  **1999**: 10.9 (7.1–16.9)  **2000**: 17.7 (13.3–23.6)  **2001**: 18.4 (14.1–23.9)  **2002**: 13.9 (10.7–18.0)  **2003**: 16.5 (13.3–20.4)  **2004**: 16.6 (13.5–20.3)  **2005**: 13.7 (11.2–16.8)  **2006**: 14.5 (12.1–17.4)  **2007**: 13.5 (11.2–16.3)  **2008**: 13.6 (11.4–16.4) |
| Eylert et al. 2013 | ASR (95% CI):  Total  **1985-87**: 0.86 (0.81-0.90)  **1986-88**: 0.81 (0.76-0.85)  **1987-89**: 0.83 (0.78-0.88)  **1988-90**: 0.86 (0.81-0.90)  **1989-91**: 0.88 (0.83-0.92)  **1990-92**: 0.86 (0.82-0.91)  **1991-93**: 0.85 (0.80-0.89)  **1992-94**: 0.87 (0.82-0.92)  **1993-95**: 0.91 (0.86-0.95)  **1994-96**: 0.93 (0.89-0.98)  **1995-97**: 0.98 (0.93-1.03)  **1996-98**: 1.01 (0.96-1.05)  **1997-99**: 0.99 (0.95-1.04)  **1998-00**: 1.00 (0.95-1.05)  **1999-01**: 0.99 (0.94-1.04)  **2000-02**: 1.02 (0.97-1.07)  **2001-03**: 1.03 (0.98-1.07)  **2002-04**: 1.04 (0.99-1.09)  **2003-05**: 1.09 (1.05-1.14)  **2004-06**: 1.15 (1.10-1.20)  **2005-07**: 1.20 (1.15-1.25)  **2006-08**: 1.23 (1.18-1.28)  **2007-09**: 1.27 (1.22-1.32)  Male  **1985-87**: 1.25 (1.17-1.33)  **1986-88**: 1.15 (1.07-1.23)  **1987-89**: 1.16 (1.09-1.24)  **1988-90**: 1.17 (1.09-1.25)  **1989-91**: 1.21 (1.13-1.29)  **1990-92**: 1.21 (1.13-1.29)  **1991-93**: 1.18 (1.11-1.26)  **1992-94**: 1.19 (1.11-1.27)  **1993-95**: 1.23 (1.15-1.31)  **1994-96**: 1.26 (1.18-1.34)  **1995-97**: 1.31 (1.23-1.40)  **1996-98**: 1.34 (1.26-1.43)  **1997-99**: 1.34 (1.26-1.42)  **1998-00**: 1.35 (1.27-1.43)  **1999-01**: 1.35 (1.27-1.43)  **2000-02**: 1.40 (1.32-1.48)  **2001-03**: 1.42 (1.34-1.50)  **2002-04**: 1.43 (1.35-1.51)  **2003-05**: 1.49 (1.41-1.57)  **2004-06**: 1.56 (1.48-1.65)  **2005-07**: 1.63 (1.54-1.71)  **2006-08**: 1.66 (1.58-1.74)  **2007-09**: 1.72 (1.64-1.81)  Female  **1985-87**: 0.46 (0.42-0.51)  **1986-88**: 0.46 (0.42-0.51)  **1987-89**: 0.49 (0.45-0.54)  **1988-90**: 0.54 (0.49-0.59)  **1989-91**: 0.55 (0.50-0.60)  **1990-92**: 0.52 (0.47-0.56)  **1991-93**: 0.51 (0.46-0.55)  **1992-94**: 0.55 (0.50-0.60)  **1993-95**: 0.58 (0.53-0.63)  **1994-96**: 0.61 (0.56-0.66)  **1995-97**: 0.64 (0.59-0.69)  **1996-98**: 0.67 (0.62-0.72)  **1997-99**: 0.65 (0.60-0.70)  **1998-00**: 0.65 (0.60-0.70)  **1999-01**: 0.63 (0.58-0.68)  **2000-02**: 0.64 (0.59-0.69)  **2001-03**: 0.63 (0.58-0.68)  **2002-04**: 0.65 (0.60-0.70)  **2003-05**: 0.70 (0.65-0.75)  **2004-06**: 0.74 (0.69-0.79)  **2005-07**: 0.78 (0.72-0.83)  **2006-08**: 0.80 (0.75-0.86)  **2007-09**: 0.82 (0.77-0.87) |
| Antoni et al. 2014 | ASR (for renal pelvis cancer):  Men, **1983-1987**  All 5 registries: 2.02  Men, **2003-2007**  All 5 registries: 1.24  Women, **1983-1987**  All 5 registries: 2.26  Women, **2003-2007**  All 5 registries: 1.08 |
| Cauberg et al. 2010 | Incidence rate  **1995**: 2.10 per 100,000 person-years  **2003**: 2.40 per 100,000 person-years  (extracted using WebPlotDigitizer)  **1996**: 2.30  **1997**: 2.08  **1998**: 2.37  **1999**: 2.26  **2000**: 2.35  **2001**: 2.24  **2002**: 2.32 |
| Woodford et al. 2016 | ASR:  Overall (males and females)  **2001**: 1.20347  **2002**: 1.36894  **2003**: 1.04255  **2004**: 1.34059  **2005**: 1.41106  **2006**: 1.41782  **2007**: 1.39842  **2008**: 1.57944  **2009**: 1.1961  **2010**: 1.45674  **2011**: 1.33985  Males  **2001**: 1.45542  **2002**: 1.56509  **2003**: 1.06538  **2004**: 1.60044  **2005**: 1.53182  **2006**: 1.46722  **2007**: 1.64522  **2008**: 1.88928  **2009**: 1.23574  **2010**: 1.5808  **2011**: 1.37193  Females  **2001**: 0.58113  **2002**: 0.70593  **2003**: 0.92708  **2004**: 0.86769  **2005**: 0.77873  **2006**: 1.117  **2007**: 0.67735  **2008**: 0.73902  **2009**: 0.79869  **2010**: 0.72352  **2011**: 0.99595 |
| Mellemgaard et al. 1993 | Incidence rate per 100,000 person-years: (extracted using WebPlotDigitizer)  Ureter  **1945**: 0.16  **1950**: 0.18  **1955**: 0.45  **1960**: 0.55  **1965**: 0.72  **1970**: 1.20  **1975**: 1.74  **1980**: 1.67  **1985**: 1.67  Pelvis  **1945**: 0.09  **1950**: 0.12  **1955**: 0.18  **1960**: 0.25  **1965**: 0.46  **1970**: 0.57  **1975**: 0.68  **1980**: 0.66  **1985**: 0.89 |
| Munoz et al. 2000 | ASR per 100,000 person-years: (extracted using WebPlotDigitizer)  Pelvis  **1973-1975**: 1.01  **1976-1978**: 1.12  **1979-1981**: 1.06  **1982-1984**: 1.05  **1985-1987**: 1.02  **1988-1990**: 1.04  **1991-1993**: 1.0  **1994-1996**: 1.04  Ureter  **1973-1975**: 0.6  **1976-1978**: 0.66  **1979-1981**: 0.75  **1982-1984**: 0.73  **1985-1987**: 0.73  **1988-1990**: 0.75  **1991-1993**: 0.72  **1994-1996**: 0.71 |
| Lynch and Cohen 1995 | ASR per 100,000 persons (ureter):  Male  **1973-1977**: 0.8  **1978-1982**: 0.8  **1983-1997**: 0.8  Female  **1973-1977**: 0.3  **1978-1982**: 0.3  **1983-1997**: 0.8 |
| Michalek et al. 2019 | SIR (95% CI):  Year of diagnosis  **1961-1975**  Clerical workers: 1.18 (0.97-1.41)  Electrical workers: 1.23 (0.88-1.66)  Food workers: 1.64 (1.26-2.10)  Forestry workers: 0.30 (0.15-0.52)  Gardeners: 0.72 (0.54-0.95)  Packers: 1.52 (1.21-1.88)  Painters: 0.84 (0.53-1.28)  Printers: 1.36 (0.83-2.11)  Public safety workers: 0.61 (0.31-1.06)  Seamen: 1.58 (1.02-2.36)  Textile workers: 1.17 (0.88-1.54)  Transport workers: 1.18 (0.80-1.69)  Welders: 0.95 (0.41-1.86)  Woodworkers: 0.77 (0.61-0.96)  **1976-1990**  Clerical workers: 1.24 (1.14-1.35)  Electrical workers: 1.07 (0.90-1.27)  Food workers: 1.20 (1.03-1.39)  Forestry workers: 0.59 (0.45-0.76)  Gardeners: 0.63 (0.53-0.74)  Packers: 1.30 (1.13-1.50)  Painters: 1.25 (1.01-1.52)  Printers: 1.36 (1.06-1.71)  Public safety workers: 1.39 (1.13-1.69)  Seamen: 1.65 (1.33-2.02)  Textile workers: 1.22 (1.06-1.41)  Transport workers: 1.17 (0.97-1.41)  Welders: 1.44 (1.04-1.95)  Woodworkers: 0.77 (0.67-0.87)  **1991-2005**  Clerical workers: 1.13 (1.05-1.22)  Electrical workers: 1.25 (1.09-1.43)  Food workers: 1.02 (0.86-1.19)  Forestry workers: 0.42 (0.30-0.59)  Gardeners: 0.81 (0.70-0.93)  Packers: 1.03 (0.87-1.22)  Painters: 1.31 (1.07-1.59)  Printers: 1.42 (1.14-1.75)  Public safety workers: 1.49 (1.24-1.78)  Seamen: 1.36 (1.10-1.67)  Textile workers: 1.23 (1.07-1.42)  Transport workers: 1.23 (1.04-1.45)  Welders: 1.40 (1.07-1.80)  Woodworkers: 0.86 (0.76-0.98) |
| Wu et al. 2020 | ASR (per 100,000 persons): (extracted using WebPlotDigitizer)  Overall  **1988**: 1.57  **1989**: 1.66  **1990**: 1.62  **1991**: 1.47  **1992**: 1.58  **1993**: 1.41  **1994**: 1.54  **1995**: 1.54  **1996**: 1.44  **1997**: 1.46  **1998**: 1.58  **1999**: 1.45  **2000**: 1.4  **2001**: 1.41  **2002**: 1.4  **2003**: 1.52  **2004**: 1.53  **2005**: 1.41  **2006**: 1.58  **2007**: 1.57  **2008**: 1.7  **2009**: 1.57  **2010**: 1.48  **2011**: 1.53  **2012**: 1.41  **2013**: 1.49  **2014**: 1.48  **2015**: 1.51  Male  **1988**: 2.44  **1989**: 2.41  **1990**: 2.5  **1991**: 2.02  **1992**: 2.23  **1993**: 1.99  **1994**: 2.0  **1995**: 2.17  **1996**: 2.01  **1997**: 2.04  **1998**: 2.23  **1999**: 1.97  **2000**: 2.07  **2001**: 1.98  **2002**: 2.04  **2003**: 2.25  **2004**: 2.1  **2005**: 1.95  **2006**: 2.03  **2007**: 2.26  **2008**: 2.22  **2009**: 2.04  **2010**: 2.12  **2011**: 2.13  **2012**: 2.02  **2013**: 1.95  **2014**: 1.99  **2015**: 2.07  Female  **1988**: 0.97  **1989**: 1.14  **1990**: 1.01  **1991**: 1.07  **1992**: 1.10  **1993**: 0.98  **1994**: 1.17  **1995**: 1.07  **1996**: 1.05  **1997**: 1.05  **1998**: 1.13  **1999**: 1.04  **2000**: 0.94  **2001**: 0.99  **2002**: 0.94  **2003**: 1.02  **2004**: 1.11  **2005**: 1.04  **2006**: 1.22  **2007**: 1.05  **2008**: 1.32  **2009**: 1.22  **2010**: 0.98  **2011**: 1.06  **2012**: 0.93  **2013**: 1.13  **2014**: 1.10  **2015**: 1.07  60-69 years  **1988**: 5.66  **1989**: 6.11  **1990**: 4.64  **1991**: 4.04  **1992**: 5.17  **1993**: 4.89  **1994**: 4.53  **1995**: 4.84  **1996**: 4.33  **1997**: 5.21  **1998**: 5.14  **1999**: 4.36  **2000**: 4.89  **2001**: 4.84  **2002**: 3.76  **2003**: 4.29  **2004**: 4.78  **2005**: 4.49  **2006**: 5.11  **2007**: 3.88  **2008**: 4.69  **2009**: 4.32  **2010**: 3.19  **2011**: 4.21  **2012**: 3.43  **2013**: 3.48  **2014**: 3.75  **2015**: 3.50  70+ years  **1988**: 9.86  **1989**: 10.22  **1990**: 11.29  **1991**: 9.84  **1992**: 10.78  **1993**: 9.04  **1994**: 9.99  **1995**: 9.84  **1996**: 9.75  **1997**: 10.09  **1998**: 10.71  **1999**: 10.09  **2000**: 9.41  **2001**: 9.61  **2002**: 10.21  **2003**: 11.28  **2004**: 11.22  **2005**: 9.73  **2006**: 11.32  **2007**: 11.76  **2008**: 12.58  **2009**: 11.79  **2010**: 11.66  **2011**: 11.11  **2012**: 10.77  **2013**: 11.62  **2014**: 11.70  **2015**: 11.89  Renal pelvis  **1988**: 0.98  **1989**: 1.00  **1990**: 0.98  **1991**: 0.93  **1992**: 0.97  **1993**: 0.89  **1994**: 1.00  **1995**: 0.98  **1996**: 0.89  **1997**: 0.88  **1998**: 1.00  **1999**: 0.86  **2000**: 0.88  **2001**: 0.81  **2002**: 0.87  **2003**: 0.93  **2004**: 0.94  **2005**: 0.82  **2006**: 0.85  **2007**: 0.97  **2008**: 1.03  **2009**: 1.01  **2010**: 0.93  **2011**: 0.91  **2012**: 0.93  **2013**: 0.95  **2014**: 0.97  **2015**: 0.90  Ureter  **1988**: 0.58  **1989**: 0.65  **1990**: 0.64  **1991**: 0.54  **1992**: 0.61  **1993**: 0.52  **1994**: 0.53  **1995**: 0.55  **1996**: 0.55  **1997**: 0.58  **1998**: 0.58  **1999**: 0.57  **2000**: 0.51  **2001**: 0.60  **2002**: 0.52  **2003**: 0.58  **2004**: 0.58  **2005**: 0.58  **2006**: 0.72  **2007**: 0.59  **2008**: 0.66  **2009**: 0.55  **2010**: 0.54  **2011**: 0.61  **2012**: 0.47  **2013**: 0.53  **2014**: 0.51  **2015**: 0.60 |
| Tempo et al. 2020 | ASR (per 100,000 persons): (extracted using WebPlotDigitizer)  **1977**: 1.51  **1978**: 2.14  **1979**: 2.55  **1980**: 2.77  **1981**: 2.89  **1982**: 1.23  **1983**: 3.36  **1984**: 1.92  **1985**: 2.25  **1986**: 2.28  **1987**: 1.95  **1988**: 1.80  **1989**: 1.55  **1990**: 1.96  **1991**: 2.17  **1992**: 2.13  **1993**: 1.23  **1994**: 1.90  **1995**: 1.44  **1996**: 2.26  **1997**: 1.64  **1998**: 1.46  **1999**: 1.69  **2000**: 1.48  **2001**: 1.85  **2002**: 0.89  **2003**: 1.80  **2004**: 1.93  **2005**: 1.79  **2006**: 2.20  **2007**: 2.43  **2008**: 1.97  **2009**: 2.42  **2010**: 1.89  **2011**: 2.18  **2012**: 1.80  **2013**: 2.24 |
| Almås et al. 2021 | ASR (per 100,000 persons):  **Year**  **1999**: 3.00  **2000**: 3.08  **2001**: 2.83  **2002**: 3.76  **2003**: 3.39  **2004**: 3.61  **2005**: 2.74  **2006**: 3.32  **2007**: 4.34  **2008**: 4.07  **2009**: 4.23  **2010**: 4.17  **2011**: 3.82  **2012**: 3.66  **2013**: 4.04  **2014**: 4.16  **2015**: 4.70  **2016**: 5.39  **2017**: 4.38  **2018**: 4.89  **1999-2003**: 3.21  **2004-2008**: 3.62  **2009-2013**: 3.98  **2014-2018**: 4.70 |

# Studies by occupation

| Study | Values reported |
| --- | --- |
| Hsiao et al. 2016 | Incidence density (per 10,000 person-years):  No hemodialysis  **Occupation**  **White collar**: 0.67  **Blue collar**: 0.70  **Others**: 0.56  Yes hemodialysis  **Occupation**  **White collar**: 14.6  **Blue collar**: 27.7  **Others**: 18.4 |
| Pukkala et al. 2009 | SIR (95% CI):  Men  **Seamen**: 1.52 (95% CI 1.26-1.85)  **Printers**: 1.39 (1.09-1.74)  **Welders**: 1.39 (1.05-1.80)  **Public safety workers**: 1.34 (1.12-1.61)  **Textile workers**: 1.30 (1.04-1.61)  **Forestry workers**: 0.48 (0.36-0.62)  **Farmers**: 0.60 (0.55-0.66)  Women  **Clerical workers**: 1.19 (1.08-1.31)  **Shop workers**: 1.16 (1.04-1.31)  **Religious workers**: 0.53 (0.29-0.89)  **Farmers**: 0.57 (0.45-0.72)  **Gardeners**: 0.66 (0.50-0.86) |
| McLaughlin et al. 2012 | SIR (for renal pelvis) (95% CI):  Total: 0.78 (0.34-1.54) |
| McLaughlin et al. 1987 | SIR (for renal pelvis):  **General industry**  **Farming, forestry, hunting, and fishing**: 0.67  **Mining and quarrying**: 1.31  **Manufacturing I**: 1.05  **Manufacturing II**: 1.22  **Construction**: 1.07  **Electric, gas, water, and sanitary services**: 1.07  **Trade, finance, insurance, and real estate**: 1.01  **Transport and communication**: 1.11  **Services**: 1.14  **Non-classifiable services**: 0.41  **Food products**: 1.41  **Tobacco and beverages**: 1.08  **Textile**: 0.84  **Apparel**: 1.25  **Lumber and wood products**: 0.70  **Furniture and fixtures**: 0.94  **Paper and allied products**: 1.12  **Printing and publishing**: 1.02  **Leather**: 1.23  **Rubber**: 1.10  **Chemical**: 0.74  **Coal and petroleum refining**: 1.16  **Stone, clay, glass**: 1.10  **Fabricated metal products**: 1.17  **Machinery and electronics**: 1.42  **Transportation equipment**: 0.98  **Miscellaneous manufacturing**: 2.15  **Manufacturing, NEC**: -  **Home building construction**: 0.95  **Other construction**: 1.20  **Other ore mining**: 4.49  **Machine industry**: 1.60  **Scientific and surgical instrument construction**: 5.18  **Plumbing installation**: 2.37  **Legal services**: 4.67  **Theatres**: 5.23  **Specific occupation**  **Professional, technical, and related workers**: 1.17  **Administrative, executive, and managerial workers**: 0.93  **Clerical workers**: 1.13  **Sales workers**: 1.14  **Farmers, fishermen, hunters, and related workers**: 0.66  **Miners, quarrymen, and related workers**: 1.45  **Transport and communication workers**: 0.94  **Craftsmen, production workers, and labourers**: 1.08  **Craftsmen, production workers, and labourers, NEC**: 1.22  **Services, sport, and recreation workers**: 1.18  **Spinners, weavers, and knitters**: 0.63  **Tailors, cutters, furriers**: 1.12  **Shoe and leather workers**: 1.07  **Metal making and treating workers**: 1.34  **Precision instrument workers**: 1.11  **Toolmakers, machinists, plumbers, welders**: 1.31  **Electricians, electronic workers**: 1.29  **Woodworkers**: 0.75  **Painters, paperhangers**: 0.69  **Bricklayer and construction worker, NEC**: 1.08  **Graphical worker**: 1.50  **Potters, kilnmen, and glass workers**: 2.12  **Food industry workers**: 1.25  **Chemical and cellulose workers**: 1.15  **Tobacco workers**: 1.09  **Craftsmen and production process workers: 0.99**  **Labourers, NEC**: 1.07  **Stationary engine and equipment operators**: 1.38  **Engineers and technicians in mining and metallurgy**: 3.29  **Judges**: 9.16  **Wholesale buyers**: 1.91  **Machinists and toolmakers**: 1.52  **Plumbers**: 2.17 |
| Lynge et al. 1995 | SIR (95% CI):  **Typographer/printing establishments**: 2.28 (0.83-4.97)  **Typographer/newspapers/magazines**: 2.16 (0.58-5.54)  **Typographer/all other**: -  **Lithographer**: 3.39 (0.38-12.24)  **Photoengraver**: -  **Bookbinder**: -  **Factory worker/all printing industry**: 1.80 (0.36-5.25)  **All groups**: 1.89 (1.06-3.11)  **Electrician**: 1.16 (0.37-2.70)  **Painter**: 1.04 (0.42-2.14)  **Bricklayer**: 1.39 (0.69-2.48)  **Metal worker**: 1.35 (1.00-1.79)  **Postal worker**: 0.82 (0.27-1.92)  **Unskilled worker**: 1.00 (0.89-1.12) |
| Michalek et al. 2019 | SIR (95% CI):  **Occupational category**  **Administrators**: 1.09 (0.98-1.20)  **Artistic workers**: 1.31 (0.97-1.74)  **Assistant nurses**: 1.07 (0.85-1.32)  **Beverage workers**: 1.05 (0.59-1.73)  **Bricklayers**: 1.03 (0.79-1.31)  **Building caretakers**: 0.97 (0.87-1.09)  **Chemical process workers**: 1.09 (0.88-1.33)  **Chimney sweeps**: 1.73 (0.70-3.57)  **Clerical workers**: 1.18 (1.09-1.27)  **Cooks and stewards**: 0.81 (0.58-1.10)  **Dentists**: 1.13 (0.66-1.80)  **Domestic assistants**: 0.94 (0.77-1.13)  **Drivers**: 1.01 (0.91-1.13)  **Economically inactive**: 0.97 (0.93-1.00)  **Electrical workers**: 1.18 (1.02-1.36)  **Engine operators**: 0.85 (0.70-1.03)  **Farmers**: 0.61 (0.56-0.66)  **Fishermen**: 0.84 (0.64-1.08)  **Food workers**: 1.16 (1.01-1.34)  **Forestry workers**: 0.47 (0.35-0.62)  **Gardeners**: 0.72 (0.62-0.83)  **Glass makers, etc.**: 1.08 (0.90-1.29)  **Hairdressers**: 1.19 (0.86-1.60)  **Journalists**: 1.15 (0.69-1.80)  **Laboratory assistants**: 1.20 (0.70-1.92)  **Launderers**: 0.99 (0.70-1.34)  **Mechanics**: 1.13 (1.04-1.24)  **Military personnel**: 1.00 (0.74-1.32)  **Miners and quarry workers**: 1.02 (0.68-1.47)  **Nurses**: 1.05 (0.81-1.33)  **Other construction workers**: 0.88 (0.76-1.01)  **Other health workers**: 1.17 (0.92-1.47)  **Other workers**: 1.09 (0.98-1.21)  **Packers**: 1.23 (1.07-1.41)  **Painters**: 1.22 (1.00-1.46)  **Physicians**: 1.24 (0.90-1.68)  **Plumbers**: 1.26 (0.97-1.62)  **Postal workers**: 1.02 (0.84-1.23)  **Printers**: 1.39 (1.11-1.71)  **Public safety workers**: 1.35 (1.12-1.62)  **Religious workers, etc.**: 0.88 (0.72-1.07)  **Sales agents**: 1.10 (0.98-1.24)  **Seamen**: 1.51 (1.23-1.82)  **Shoe and leather workers**: 1.26 (0.90-1.72)  **Shop workers**: 1.12 (1.03-1.22)  **Smelting workers**: 1.13 (0.94-1.34)  **Teachers**: 0.95 (0.84-1.07)  **Technical workers, etc.**: 1.09 (1.00-1.19)  **Textile workers**: 1.22 (1.06-1.39)  **Tobacco workers**: 1.28 (0.55-2.53)  **Transport workers**: 1.20 (1.01-1.42)  **Waiters**: 1.07 (0.81-1.39)  **Welders**: 1.37 (1.03-1.78)  **Woodworkers**: 0.81 (0.72-0.91) |
| Michalek et al. 2019 | SIR (95% CI):  Non-adjusted for smoking  **Dentists**: 1.31 (0.73–2.16)  **Physicians**: 1.30 (0.93–1.78)  **Chimney sweeps**: 1.75 (0.70–3.60)  **Artistic workers**: 1.37 (0.99–1.86)  **Other health workers**: 1.30 (0.89–1.85)  **Public safety workers**: 1.35 (1.11–1.62)  **Laboratory assistants**: 1.27 (0.58–2.41)  **Textile workers**: 1.30 (1.04–1.61)  **Printers**: 1.37 (1.08–1.73)  **Transport workers**: 1.21 (1.02–1.43)  **Welders**: 1.39 (1.05–1.80)  **Clerical workers**: 1.15 (1.02–1.30)  **Seamen**: 1.51 (1.23–1.82)  **Assistant nurses**: 1.10 (0.50–2.09)  **Technical workers**: 1.08 (0.99–1.18)  **Administrators**: 1.08 (0.97–1.19)  **Religious workers**: 0.98 (0.79–1.20)  **Electrical workers**: 1.16 (0.99–1.35)  **Plumbers**: 1.26 (0.97–1.62)  **Packers**: 1.24 (1.06–1.44)  **Painters**: 1.22 (1.00–1.47)  **Food workers**: 1.18 (0.99–1.38)  **Gardeners**: 0.75 (0.63–0.89)  **Miners and quarry workers**: 1.03 (0.68–1.48)  **Woodworkers**: 0.82 (0.72–0.92)  **Engine operators**: 0.85 (0.69–1.03)  **Fishermen**: 0.83 (0.63–1.07)  **Unskilled construction workers**: 0.88 (0.76–1.01)  **Farmers**: 0.62 (0.56–0.67)  **Waiters**: 0.94 (0.47–1.69)  **Beverage workers**: 0.75 (0.32–1.47)  **Launderers**: 0.65 (0.26–1.34)  **Cooks and stewards**: 0.69 (0.33–1.27)  **Forestry workers**: 0.48 (0.36–0.62)  Adjusted for smoking  **Dentists**: 1.66 (0.93–2.74)  **Physicians**: 1.63 (1.16–2.23)  **Chimney sweeps**: 1.47 (0.59–3.02)  **Artistic workers**: 1.43 (1.03–1.94)  **Other health workers**: 1.42 (0.96–2.01)  **Public safety workers**: 1.38 (1.14–1.65)  **Laboratory assistants**: 1.35 (0.62–2.57)  **Textile workers**: 1.32 (1.05–1.63)  **Printers**: 1.28 (1.01–1.61)  **Transport workers**: 1.24 (1.05–1.47)  **Welders**: 1.23 (0.93–1.60)  **Clerical workers**: 1.21 (1.07–1.37)  **Seamen**: 1.21 (0.99–1.47)  **Assistant nurses**: 1.18 (0.54–2.24)  **Technical workers**: 1.18 (1.08–1.29)  **Administrators**: 1.17 (1.05–1.30)  **Religious workers**: 1.17 (0.95–1.44)  **Electrical workers**: 1.16 (0.99–1.35)  **Plumbers**: 1.09 (0.83–1.40)  **Packers**: 1.12 (0.96–1.29)  **Painters**: 1.12 (0.92–1.35)  **Food workers**: 1.10 (0.93–1.30)  **Gardeners**: 0.87 (0.73–1.03)  **Miners and quarry workers**: 0.84 (0.56–1.21)  **Woodworkers**: 0.84 (0.74–0.94)  **Engine operators**: 0.79 (0.65–0.96)  **Fishermen**: 0.79 (0.60–1.02)  **Unskilled construction workers**: 0.78 (0.68–0.90)  **Farmers**: 0.76 (0.69–0.83)  **Waiters**: 0.70 (0.35–1.25)  **Beverage workers**: 0.64 (0.28–1.27)  **Launderers**: 0.59 (0.24–1.21)  **Cooks and stewards**: 0.57 (0.27–1.05)  **Forestry workers**: 0.51 (0.38–0.66) |

# Lynch syndrome

| Study | Values reported |
| --- | --- |
| Ericson et al. 2004 | SIR (95% CI):  **HNPCC risk groups**  All: 1.2 (0.9-1.5)  Onset ≥50y: 1.0 (0.7-1.4)  Onset <50: 1.0 (0.0-5.6)  Multiple, onset ≥50: 1.8 (0.2-6.6)  Familial, onset ≥50: 11.2 (2.3-32.0)  Multiple, onset <50: 0.0 (0.0-147.0)  Familial, onset <50: 29.6 (8.1-75.9)  **Type of malignancy in parent**  Colon  Diagnosis ≥50: 0.9 (0.5-1.4)  Diagnosis <50: 2.9 (0.07-16.0)  Rectal  Diagnosis ≥50: 1.4 (0.8-2.3)  Diagnosis <50: 0  Endometrial  Diagnosis ≥50: 1.6 (0.8-2.8)  Diagnosis <50: 0  Small intestine  Diagnosis ≥50: 2.1 (0.05-11.9)  Diagnosis <50: 0  Upper urinary tract  Diagnosis ≥50: 2.0 (0.5-5.2)  Diagnosis <50: 0 |
